# Association of multi-morbidity, social participation, functional and mental health with the self-rated health of middle-aged and older adults in India: a study based on LASI wave-1

**DOI:** 10.1186/s12877-022-03349-0

**Published:** 2022-08-16

**Authors:** Gursimran Singh Rana, Anandi Shukla, Akif Mustafa, Mahadev Bramhankar, Balram Rai, Mohit Pandey, Nand Lal Mishra

**Affiliations:** International Institute for Population Sciences (IIPS), Mumbai, 400088 India

**Keywords:** SRH, Elderly health, Mental health, Functional health, Social participation index, Older adults

## Abstract

Self-rated health (SRH) is a well-established measure in public health to administer the general health of an individual. It can also be used to assess overall health status’ relationship with the social, physical, and mental health of a person. In this study, we examine the association of SRH and various socio-economic & health-related factors such as multi-morbidity status, mental health, functional health, and social participation. Data used in this paper is collated from the first wave of Longitudinal Ageing Study in India (LASI) 2017-18. A total of 65,562 older adults aged 45 or above are considered in our study. Various indices (multimorbidity, social participation, functional and mental health) have been created to measure factors influencing the SRH of an individual. Overall, in the study population, around 18.4% of people reported poor SRH. Dominance Analysis results show that the contribution of multimorbidity in predicting poor SRH is highest, followed by functional health, mental health, and social participation. In a developing country like India, there is a dire need for policies having a holistic approach regarding the health and well-being of the older population.

## Introduction

Self-rated health (SRH) which is also known as self-perceived health, is an easy preferable, and simple measure to administer an individual’s overall health [1]. It is generally referred to as a single research question in the survey, that is, “How would you rate your overall health” with the response items “Very poor”, “Poor”, “Fair”, “Good,” and “Very good”. According to World Health Organisation (WHO), SRH is an important indicator of population health and healthy life expectancy [2]. Kaplan et al. stated SRH as a social construct [3]. It has resulted from a complex process of cognitive-emotional well-being of individuals established by the community and culture they live and follow and, can be understood as summarised measure of individual level factors that can vary a lot [1]. The SRH is considered a subjective measure and in numerous studies it is found to be highly correlated with other health measures [4–8]. It can also predict individual’s future health problems and their need for health care, as well as successful ageing and survival [9–13].

Further, other existing studies revealed that the SRH is a well-established measure in public health to administer the general health of an individual. The SRH measure has high reliability and criterion validity [14, 15] and can also be used to assess overall health status’ relationship with the social, physical, and mental health of a person [16, 17]. Therefore, SRH has been reported as one of the vital measures available to measure health outcomes [18].

According to the census 2011, the Indian older population aged 60 or above is 8.6%, accounting for 103 million elderly population [19]. By 2050 the Indian elderly population will reach around 319 million, approximately 20% share of the total population. On inclusion of the pre-retirement phase population (aged 45 and above), this share will rise to 40% of the total population [20]. Older adults play an essential role at the family and societal levels. However, with the rising older population, many concerns and vulnerabilities will also increase for this specific population. According to WHO-SAGE, every Indian older adult suffers from at least one chronic disease, which depicts the high NCDs burden in older adults [21]. Around 12.5% of middle-aged and older adults (45 and above) are found to be suffering from multi-morbidity, which has been proven to be directly associated with adverse health outcomes such as reduced physical functions [22], lower quality of life [23], poor SRH [24] and increased mortality [25]. These demographic changes will require swift actions to cope with upcoming health, economic and societal changes. Hence, it is essential to focus on the factors that determine the health of older adults and ensure their physical and mental well-being.

Although in recent times SRH has replaced clinical assessment measures in surveys, yet we only have little knowledge about how individuals form their perspective of SRH and what factors are associated with poor SRH rating, especially in the elderly population. Various individual and socio-economic factors are found to play significant role in determining SRH and have direct association with it [25–30]. Social support is linked to less stress, positive health behaviours, and a sense of security [31]. Any perceived social or financial support tends to influence the SRH of older people. According to the literature, mental and physical health are also strongly associated with SRH among the elderly [25, 32, 33].

Various disciplines have studied the determinants of SRH, but not much research has been done on the older Indian population which is increasing substantially. As we know, SRH is not only determined by physical health, age, etc.; other psycho-social factors also play a vital role in its determination. Several studies have thoroughly explored the relationship between SRH and physical health indicators such as multimorbidity, functional health and mental health. However, the association between SRH and social participation has received little attention. India has a rich sociological structure where emotional and social bonds play a vital role specially in the older age. Thus, social participation becomes more important indicator in shaping health of older adults especially in Indian context. In this study, we examined the association of SRH and various socio-economic & health-related factors such as multi-morbidity status, mental health, functional health, and social participation. We have also analysed the relative contribution of these factors in explaining the variation in SRH among the Indian older adults aged 45 or above, to have better understanding in terms of need to prioritise the factors for the improvement of overall health and well-being of study population. This study attempts to provide a more comprehensive picture of SRH, providing more clear insights of its associated factors in Indian population.

## Data and methods

### Data

Data used in this paper is collated from the first wave of Longitudinal Ageing Study in India (LASI) conducted in 2017-18. The LASI is a nation-wide survey dedicated to health, economic, social determinants and consequences of population ageing in India [20]. The first wave of the survey covered 72,250 older adults aged 45 and above and their spouses in India across all states and union territories except Sikkim applying a multistage stratified area probability cluster sampling design for the selection of the participants. The overall response rate of the survey was 87.3% ranging from 74.3% in Chandigarh to 96.3% in Nagaland. Detailed information on the survey design, instruments used, and data collection can be accessed from LASI India Report [20]. The present study focuses on the middle-aged and older adults aged 45 years and above. The total sample size for this study is 65,562 individuals of age 45 years and above.

### Outcome variable

The SRH of the respondents was assessed based on the question ‘Overall, how is your health in general? Would you say it is very good, good, fair, poor, or very poor? In our analyses we have used the dichotomized version of this variable where “poor”, and “very poor”, were coded as “Yes”, and “very good” “good”, and “fair” were coded as “No” for poor SRH [34, 35].

### Explanatory variables

Explanatory variables included in this analysis are background characteristics, health risk factors including physical activity, tobacco, and alcohol consumption, presence of multi-morbidity, functional limitations, mental health conditions, social participation, and self-rated life satisfaction. Background characteristics include the place of residence (rural and urban), age (45-59, 60-74 and 75+ years), sex (male and female), marital status (currently married, widowed, and divorced/separated/deserted/others), living arrangement (living alone, living with spouse and/or others, living with spouse & children, living with children & others, and living with others only), education level (respondents with- no schooling, less than 5 years of schooling, 5 to 9 years of schooling and 10 or more years of schooling), work status (worked in the past but currently not working, never worked and currently working), religion (Hindu, Muslim, Christian, and Others), caste/tribe (scheduled tribes, scheduled caste, other backward classes, and others), monthly per capita expenditure (poorest, poorer, middle, richer and richest quintiles), and geographical regions (north, south, east, west, north-east and central regions).

Health risk behaviours included are using tobacco (coded as never used, currently using and used in the past), drinking alcohol (ever drank alcohol coded as yes and no), and physically active (coded as active and inactive). Physical activity was assessed through questions under two heads- moderate and vigorous physical activities. Moderate physical activity includes engagement of respondents in cleaning the house, washing clothes, drawing water from a well, fetching water, gardening, bicycling at a regular pace, walking at a moderate pace, and floor or stretching exercises, whereas for vigorous activity, they were asked about their involvement in running or jogging, going to a health centre/gym, cycling, swimming, digging with a spade or shovel, chopping, farm work, heavy lifting, fast bicycling, and cycling with loads. As per WHO norms, those who were either engaged in moderate physical activity (at least 150 minutes throughout the week) or vigorous physical activity (at least 75 minutes throughout the week) or an equivalent combination of both were categorized as physically active [20].

The presence of multi-morbidity is coded as no morbidity, single morbidity, and multi-morbidity is defined as the presence of two or more morbid conditions, including hypertension, chronic heart diseases, any chronic lung disease, diabetes, any bone/joint disease, and cancer.

To assess Activities of Daily Living (ADL) limitations, LASI respondents were asked if they were having any of the following limitations and expected the limitation to last for more than 3 months: difficulty with dressing, walking across the room, bathing, eating, getting in or out of bed, or using the toilet (including getting up and down). In the LASI, Instrumental Activities of Daily Living (IADL) was assessed by asking respondents if they were having any difficulties that were expected to last for at least 3 months, such as shopping for groceries, preparing a hot meal, making a telephone call, doing work around the house or garden, taking medications, managing money like paying bills and keeping track of expenses, and getting around or finding an address in unfamiliar places. In this study, we created a combination of 1 + ADL and 1 + IADL limitations for each individual and categorized them into a person having- neither 1 + ADL nor 1 + IADL, either 1 + ADL or 1 + IADL, and both 1 + ADL as well as 1 + IADLlimitations in order to assess the functional health.

LASI used the Health and Retirement Study (HRS) cognition module to measure the cognitive impairment across five domains- memory, orientation, arithmetic function, executive function, and object naming of cognition. The lowest 10th percentile of the composite score ranging from 0 to 43 refers to ‘poor’ cognition. It is used as a proxy measure of poor cognitive functioning in this study [36]. The Composite International Diagnostic Interview (CIDI-SF) scale, a structured interview scale, was used by the survey to diagnose probable major depression. The CIDI-SF scale score ranges from 0 to 10, and a score of three or more is used to calculate the prevalence of probable major depression [37]. The mental health conditions variable in our analysis is a combination of poor cognitive health and presence of depression symptoms where 0, 1, and 2, respectively, indicate older adults with- neither poor cognitive health nor depressive symptoms, either poor cognitive health or depressive symptoms, and both poor cognitive health and depressive symptoms.

The variable of social participation is based on response to six questions asking the frequency of attending organizations, clubs, or society’s meetings/gathering; visiting relatives/friends; attending cultural performances, shows or cinema; attending religious functions/events such as bhajan, Satsang or prayer; attending political, community or organization group meetings; and meeting with friends. Frequency was coded as 0 for never attending/visiting/meeting, 1 for at least once a year, 2 for at least once a month, 3 for at least once a week, and 4 for daily. The social participation score is the sum of all these codes ranging on a scale of 0 to 24. Further, we categorized this into three equal parts, i.e., first, second and third terciles.

### Statistical analysis

We conducted the descriptive statistical analysis to examine the variation in ‘poor’ SRH by selected background characteristics, health risk behaviours, physical, functional, mental, and social health status. Further, we fit a multivariable logistic regression model to determine the adjusted effect of predictor variables on the likelihood of poor-SRH. In addition, we applied dominance analysis to quantify the relative importance of predictors of poor SRH. We selected only those variables for dominance analysis that were statistically significant in the logistic regression analyses. Dominance analysis is an extension of multiple regression popularly used in psychological research to analyse the relative importance of predictor variables in a regression model [38]. This technique first makes all possible combinations of all the predictor variables. It calculates R^2^ for each combination by running regression against the outcome variable and measuring the relative importance of predictor variables by pairwise comparison of the R^2^ values. The relative importance of the predictors is assessed based on the predictor’s percentage share in the total explained variation by the regression model. STATA-16 with ‘domin’ and ‘moremata’ packages were used to conduct statistical analysis [39].

## Results

Table 1 presents the weighted proportion of the sample reporting poor SRH and its distribution among various socio-demographic characteristics and health behaviors. It also describes the distribution of various components, i.e., multi-morbidity, functional limitations, mental conditions, and social participation, conceptualized in resulting poor SRH. The overall prevalence of poor SRH among the Indian population aged 45 above is 18.4%. The older adults in rural areas reported a higher proportion of poor SRH than the urban areas. The proportion of older adults reporting poor SRH increases with age. Female older adults have a higher proportion of poor SRH reported, i.e., 19.5% compared to 17.1% among males. With the increase in the number of years of education among adults, the proportion of people reporting poor SRH significantly declines from 21.1% in the no schooling category to 10.5% in 10 or more years of completed education category. There is not much significant difference in the proportion of poor SRH among all five quantiles of the wealth index. The widowed and separated or divorced adults report a higher proportion of poor SRH, i.e., 24.7 and 22.7%, respectively, than 16.2% among currently married older adults. The highest proportion of older adults reporting poor SRH is found in the southern region (23.3%), while the lowest is in the western region (12%). The older adults living alone reported the highest proportion of poor SRH (30.6%) among all combinations of living arrangements, including living with spouse and children (15.3%), living with children, and others (19.7%). 28.6% of older adults who have consumed tobacco in any form reported their health as poor. There is a significant difference in the proportion of older adults reporting their health as poor between physically active (14.6%) and physically inactive (25.1%) adults. 55.1% of older adults who are not at all satisfied with their lives report their health as poor compared to 13% who are completely satisfied with their lives.

**Table 1:** Prevalence (%) of Multi-morbidity, Functional Health Limitations, Mental Health Conditions, High Social participation Score and Poor SRH by Background Characteristics in India, LASI Wave-1, 2017-18

Characteristics		Multi-morbidity^**a**^			Functional Health Limitations^**b**^			Mental Health Conditions^**c**^			Social participation			SRH	Sample
		No	1	1+	Neither	Either	Both	Neither	Either	Both	Low	Middle	High	Poor	n
**Residence**	**Rural**	61.1	26.1	12.8	55.9	29.7	14.5	78.9	19.9	1.2	41.5	38.2	20.4	19.5	42,424
	**Urban**	44.7	29.9	25.4	67.0	21.1	11.9	88.4	11.0	0.6	29.8	44.6	25.6	16.1	23,138
**Age**	**45-59**	62.7	25.1	12.2	70.8	22.0	7.2	86.5	12.9	0.6	32.7	42.0	25.4	12.7	34,098
	**60-74**	50.2	28.7	21.1	52.6	31.6	15.8	80.3	18.6	1.2	39.8	40.2	20.0	21.6	24,715
	**75+**	46.3	32.3	21.4	32.0	33.1	34.9	65.3	32.3	2.5	54.2	31.8	14.0	33.7	6749
**Sex**	**Male**	59.1	25.7	15.2	68.1	20.6	11.3	87.4	12.1	0.5	32.4	39.4	28.3	17.1	30,479
	**Female**	53.4	28.6	18.0	51.9	32.4	15.7	77.2	21.4	1.4	42.5	40.8	16.7	19.5	35,083
**Education in years**	**No schooling**	59.9	26.6	13.5	49.3	33.8	17.0	72.8	25.4	1.8	46.5	38.3	15.2	21.1	30,822
	**< 5 years**	52.5	29.6	17.9	55.9	28.4	15.7	84.9	14.7	0.5	35.2	39.6	25.2	20.7	7477
	**5-9 years**	53.4	28.0	18.6	68.1	21.4	10.5	91.6	8.3	0.1	31.3	43.3	25.4	17.4	14,861
	**10 or more years**	50.1	26.9	23.0	80.1	13.3	6.6	94.2	5.8	0.0	22.2	42.2	35.6	10.5	12,402
**Caste**	**ST**	72.9	19.8	7.3	61.9	27.2	10.9	74.3	24.9	0.9	36.9	40.7	22.4	13.4	11,365
	**SC**	59.7	27.0	13.3	56.3	29.0	14.7	78.4	20.4	1.2	42.2	39.1	18.7	20.9	10,959
	**OBC**	54.7	27.2	18.1	58.8	27.9	13.4	82.9	16.1	1.1	37.5	40.7	21.8	19.3	24,629
	**NOA**	50.1	30.0	19.9	61.7	24.0	14.3	85.1	14.2	0.8	35.7	39.8	24.6	16.7	18,609
**Wealth quintile**	**Poorest**	64.0	25.6	10.4	57.1	28.2	14.7	79.0	19.6	1.4	45.2	38.6	16.3	19.0	12,941
	**Poorest**	58.6	27.9	13.5	57.1	29.3	13.6	81.2	17.8	1.1	41.1	39.7	19.3	18.2	13,190
	**Middle**	57.2	26.9	15.9	60.7	25.4	13.8	82.7	16.3	1.1	37.1	40.9	22.0	17.9	13,163
	**Rich**	52.5	28.4	19.2	58.9	28.4	12.7	84.3	15.0	0.7	33.6	42.1	24.3	18.4	13,210
	**Richest**	46.1	27.8	26.2	63.5	23.2	13.2	82.6	16.8	0.6	31.0	39.6	29.3	18.7	13,058
**Work Status**	**Currently working**	63.7	24.5	11.8	68.1	23.7	8.2	84.9	14.4	0.7	32.0	40.1	27.9	14.1	32,279
	**Worked in Past**	46.8	31.5	21.8	45.6	31.6	22.8	76.3	21.9	1.8	42.3	37.9	19.8	26.5	15,297
	**Never worked**	49.8	28.8	21.4	55.3	29.1	15.6	80.9	18.2	0.9	44.9	42.3	12.8	19.5	17,986
**Marital Status**	**Currently married**	57.6	26.7	15.7	64.7	24.3	11.1	85.5	13.9	0.6	34.6	40.9	24.5	16.2	48,769
	**Widowed**	49.8	29.7	20.5	42.7	35.6	21.8	69.8	28.0	2.2	47.3	38.1	14.7	24.7	14,592
	**Divorced/Separated/Others**	66.4	22.5	11.1	61.7	26.0	12.3	84.6	14.5	1.0	43.7	37.8	18.5	22.7	2201
**Religion**	**Hindu**	57.1	27.0	15.8	59.8	26.8	13.4	82.2	16.9	1.0	38.2	40.3	21.6	17.8	48,099
	**Muslim**	48.3	29.1	22.6	55.8	28.3	16.0	80.9	18.0	1.2	40.3	37.6	22.2	21.7	7803
	**Christian**	59.5	21.8	18.7	64.8	24.2	11.0	80.0	19.3	0.6	25.8	45.2	29.0	24.4	6536
	**Others**	51.4	31.9	16.6	56.4	29.4	14.2	80.6	18.8	0.6	33.4	41.3	25.3	16.5	3124
**Region**	**East**	58.0	26.3	15.6	55.5	26.9	17.7	80.6	18.3	1.0	39.4	45.1	15.5	19.1	11,580
	**North East**	62.3	28.4	9.3	69.5	22.6	8.0	83.8	15.3	0.9	23.3	36.2	40.6	13.3	8513
	**West**	51.4	29.8	18.8	56.7	25.5	17.8	82.2	16.6	1.2	28.1	38.5	33.4	12.0	8894
	**Central**	68.6	22.3	9.1	63.6	25.2	11.3	77.0	21.8	1.3	40.3	39.2	20.4	18.6	8907
	**North**	52.5	31.0	16.6	67.9	23.7	8.4	83.0	16.3	0.7	45.1	38.1	16.8	17.4	11,966
	**South**	47.2	28.7	24.1	55.4	32.1	12.5	86.2	13.1	0.7	39.3	38.9	21.8	23.3	15,702
**Living arrangement**	**Alone**	49.2	32.8	18.1	42.4	37.3	20.3	69.2	27.7	3.1	47.6	36.8	15.6	30.6	2313
	**Spouse &/or others**	52.8	27.8	19.4	58.8	27.0	14.3	83.4	15.7	0.9	40.6	39.5	20.0	19.7	10,358
	**Spouse & children**	59.0	26.3	14.7	66.5	23.4	10.1	86.1	13.4	0.5	32.8	41.3	25.8	15.3	37,519
	**Children & others**	50.7	28.8	20.5	44.7	34.7	20.6	72.2	26.1	1.7	45.9	39.0	15.1	22.7	12,441
	**Others only**	59.1	26.4	14.5	50.4	29.8	19.8	73.6	24.1	2.3	48.6	35.7	15.7	24.5	2931
**Tobacco use**	**Never**	53.1	28.0	18.9	59.1	27.2	13.7	82.7	16.4	0.9	39.5	40.2	20.2	18.0	41,140
	**Current user**	63.3	25.1	11.7	61.3	26.3	12.4	81.1	18.0	0.9	35.3	40.1	24.6	17.8	20,203
	**Used in Past**	46.1	32.5	21.5	49.1	29.8	21.1	77.7	19.8	2.4	32.8	40.1	27.0	28.6	3642
**Alcohol**	**No**	54.7	27.8	17.5	58.1	27.6	14.3	81.7	17.3	1.0	38.9	40.2	20.9	18.5	53,290
	**Yes**	63.3	24.2	12.5	66.0	24.2	9.8	82.9	16.1	1.0	32.1	39.9	28.0	17.9	11,718
**Physical activity**	**Active**	59.1	26.0	14.9	63.2	27.0	9.8	83.8	15.4	0.8	32.5	42.3	25.2	14.6	40,134
	**Inactive**	50.7	29.4	19.8	52.7	27.1	20.2	78.3	20.3	1.4	47.0	36.5	16.5	25.1	24,870
**Life Satisfaction**	**Completely satisfied**	54.3	28.7	17.0	71.1	19.9	9.0	89.0	10.6	0.5	30.1	40.4	29.5	13.0	8993
	**Very satisfied**	58.3	25.1	16.6	65.4	24.6	10.1	86.6	13.1	0.3	33.4	42.3	24.3	10.3	23,553
	**Somewhat satisfied**	56.4	27.9	15.8	56.7	30.0	13.4	81.1	18.0	0.9	40.7	39.9	19.4	19.8	25,958
	**Not very satisfied**	51.4	29.1	19.6	43.0	32.9	24.2	64.8	31.9	3.4	44.5	38.1	17.5	40.9	4976
	**Not at all satisfied**	51.1	29.0	20.0	37.9	29.0	33.0	49.3	44.8	6.0	50.4	34.4	15.3	55.1	1379

Note: ^a^ Multi-morbidities considered are hypertension, chronic heart diseases, any chronic lung disease, diabetes, any bone/joint disease, and cancer,

^b^ Functional health limitations include 1 + ADL and 1 + IADL, ^c^ Mental health conditions included are poor cognitive health and depressive symptoms

Table 2 describes the association of SRH with multi-morbidity, functional health limitations, mental health conditions, social participation score and life satisfaction. The adjusted odds ratio and its 95% confidence interval are reported for each category of independent variables used in the model. The odds of reporting poor SRH among older adults with more than one morbidity are 3.78 times higher [AOR: 3.78, 95% CI: (3.54,4.04)] than adults with no morbidity. The odds of reporting poor SRH also increase when the adults face functional limitations, i.e., the AOR for adults having either 1 + ADL or 1+ IADL limitations is 1.42 [95% CI: (1.34,1.51)] as compared to those having neither 1 + ADL nor 1+ IADL limitations. The likelihood of reporting poor- SRH is higher among older adults having either one [AOR: 1.74, 95% CI: (1.64,1.86)] or both [AOR: 2.77, 95% CI: (2.23,3.44)] mental health conditions. The adverse health behaviors, including consuming tobacco and alcohol, are significantly associated with poor SRH. The odds of reporting poor SRH is also higher for adults who have consumed tobacco in any form [AOR: 1.48, 95% CI: (1.33,1.64)], and ever consumed alcohol [AOR: 1.14, 95% CI (1.05,1.23)]. Physically inactive older adults are more likely to report poor SRH [AOR: 1.27, 95% CI: (1.21,1.34)].

**Table 2:** Logit model estimates to examine the effects of Multi-morbidity, Functional health limitations, Mental health conditions, social participation score on the poor-SRH of older adults in India, LASI Wave-1, 2017-18

Attributes	O.R.	***p***-value	95% C.I.	
**Multi-morbidity**				
No®				
1	2	0	1.91	2.15
More than 1	3.8	0	3.54	4.04
**Functional health limitations**				
Neither®				
Either	1.4	0	1.34	1.51
Both	3	0	2.76	3.18
**Mental health conditions**				
Neither®				
Either	1.7	0	1.64	1.86
Both	2.8	0	2.23	3.44
**Social participation score**				
Low®				
Middle	0.8	0	0.8	0.89
High	0.7	0	0.67	0.78
**Life Satisfaction**				
Completely satisfied®				
Very satisfied	0.8	0	0.77	0.92
Somewhat satisfied	1.6	0	1.48	1.76
Not very satisfied	3.7	0	3.36	4.14
Not at all satisfied	5.8	0	4.97	6.75
**Tobacco use**				
Never®				
Current user	1.2	0	1.12	1.27
Used in Past	1.5	0	1.33	1.64
**Alcohol**				
No®				
Yes	1.1	0	1.06	1.23
**Physical activity**				
Active®				
Inactive	1.3	0	1.21	1.35

Note: Regression adjusted factors by Residence, Age, Sex, Education, Caste, Wealth quintile, Marital Status, Religion, Region and Living arrangement

Table 3 presents the results for the dominant analysis. Based on the results of logistic regression analyses, a total of 11 variables were selected for dominance analysis (only variables with a *p*-value less than 0.01 were selected). The software ran a total of 2047 (2^^11^ – 1) regressions to conduct a pairwise comparison of R^2^ values. Multi-morbidity is found to be the most dominant factor in terms of explaining the variation in the SRH status. The contribution of multi-morbidity towards the total variation explained by the model is 32%. Functional health and mental health are the second and third most dominant predictors in the model, with 29% and 13% contributions, respectively. Social participation was the 5th most dominant factor in the model, which contributed around 7% in the total explained variation. It is pretty evident that all the factors conceptualized in resulting SRH dominated in explaining the total variation in the model. Besides these factors, age (9.6%), physical activity (4.1%), work status (2.4%), and smoking behaviour (1.6%) also contributed to the total variation explained.

**Table 3:** Dominance analysis results to examine the contribution of Multi-morbidity, Functional health limitations, Mental health conditions, social participation score and other variables in explaining the poor-SRH of older adults in India, LASI Wave-1, 2017-18

	Std. Domin. Stat.	Ranking
**Multi-morbidity**	31.7	1
**Functional health**	29.1	2
**Mental health**	12.8	3
**Age**	9.6	4
**Social participation**	6.9	5
**Physical activity**	4.1	6
**Work status**	2.4	7
**Smoking**	1.6	8
**Alcohol**	0.9	9
**Sex**	0.6	10
**Residence**	0.5	11

Note: Total variation explained by the model is 14% (R-square = 0.139)

## Discussion

This aim of the study was to outline the current status of SRH among the middle-aged and older adults in India and emphasizing on various factors related to it. The factors that need at most attention in order to improve the SRH among the elderly are multi-morbidity, functional limitations, mental health conditions, and social participation. It is essential to highlight that, even after adjusting for all relevant socio-demographic and health influencing characteristics such as tobacco and alcohol use, physical inactivity among the adults, the above listed factors still play a significant role in predicting the SRH. Our study clearly shows that individual-level health and socio-economic characteristics in addition to the community level social participation play an essential role in determining SRH status. Older people tend to rate their health based on factors that might differ from the younger people. In a country like India, older people are a prominent figure in society and play a key role in various household & societal activities. As a result of such arrangement, social interaction and active participation in these activities may boost their spirit and indirectly influence their self-rated health. The study describes the prevalence of multi-morbidity among various socio-demographic characteristics and health behaviours associated with poor SRH. Multi-morbidity has been significantly associated with poor SRH in Indian older adults [40] and various other countries [34, 41, 42]. Our result suggests that multi-morbidity is more prevalent among older adults residing in urban areas and with upper wealth quantiles. The dominance analysis found multi-morbidity to be the most contributing factor, i.e., 31.7% towards reporting poor SRH among older adults. Older adults most often tend to rate their health poor if they suffer from more than one illness at one time. This suggests the possibility of further research into different combinations of morbidities, in particular its contribution to SRH. The older adults suffering from multi-morbidity need to be prioritized in various healthy ageing programs and policies implemented by the government.

In our study, functional health has significantly influenced a person’s SRH, explaining approximately 30% variation in Dominance Analysis. It is similar to the findings of previous studies, where functional health is found to be a factor affecting the SRH of a person [43, 44]. A person’s functional health was assessed using the ADLs [45] and IADLs [46], which are considered as an indicator that is reliable and valid to measure a person’s ability to live without any support. Few studies have related ADLs to poor quality of life, as dependent people tend to have frequent hospitalization and risk of death [47–50]. The functional capacity to perform the IADLs and mobility of the person on day-to-day life basis tend to affect SRH [51]. In our study, a person with 1+ IADL and 1+ ADL is three times more likely to report poor SRH than a person with no functional limitation. Many longitudinal studies [52, 53] suggested that poor SRH predicts poor functional health, but there are also some exceptions [54] stating that higher the negative self-assessment of health higher its association with disability. A longitudinal study conducted in the United States [7] has shown that respondents having lower functional ability were at greater risk of declining SRH.

Our study has demonstrated ageing as a factor directly associated with mental health problems and reporting poor SRH among older adults in India. In the present study, it is also evident that the older adults who suffer from the presence of multiple mental health conditions have more prevalently reported poor SRH, which convey similar findings based on earlier existing literature [55–57]. Although multiple studies have shown the association between mental health conditions and SRH, our study results focus more on the complex and multiple covariates based on Cognitive Index and Depression (CIDI-SF scale). In our study, older people having either poor cognitive health or depression or both have a higher likelihood of reporting poor SRH, i.e., 1.7 times and 2.8 times, respectively, compared to the older person having neither of them. These findings are in accordance with the results of prior studies, conducted in different regions and populations [58–61]. Our study has also illustrated the strength of association based on Dominance Analysis, evincing mental health problems at the third position after multi-morbidity and functional health. Mental health issues are predictors of poor SRH, and the present study also provokes further discussion in this area of research.

The results suggest that older adults with higher social participation scores are less likely to report poor SRH. Our results also indicate that social participation is the fifth most important factor explaining the 6.9% variation in poor SRH. Socially isolated individuals are at an increased risk of poor health outcomes [62] because of their limited source of emotional support and information [63, 64]. Organizational memberships, trust by society, cognitive involvement, or other social participation indicators have shown critical importance in determining good SRH and providing better outcomes in terms of health [62].

### Limitations

In this study, there are a few limitations. We have dichotomized the SRH variable from a 5-point scale to conduct a binary logistic regression analysis. This is likely to result in a loss of information regarding the actual SRH. For constructing the multi-morbidity index, we have considered only six major morbidities reported in the LASI report, which are self-reported by the respondents. This may likely underestimate the actual prevalence of the morbidities. The missing cases of most of our key variables were less than 1.5%, except for mental health, in which the missing cases were approximately 11%. The mental health factor is based on the composite cognition and depression scores. The Combined Cognition score ranges from 0 to 43 and includes score for orientation (0-8), object naming (0-2), arithmetic function (0-9) and executive function (paper folding & pentagon drawing) (0-4) and total word recall (0-20). Thus, the imputation for mental health factor was highly complex, seeing the scope of this study. The indicators used for calculating the social participation scores are not exhaustive. The total variation explained by the regression model in Dominance Analysis was only 14% (R^2^ = 0.139) which is substantially low from the desired R^2^. The low model R^2^ makes it challenging to conclude the most important predictors of an outcome.

## Conclusion

This study found that around one-fifth of the Indian older population has rated their health as poor. We examined the factors predicting the poor SRH. Individuals having multi-morbidity and functional limitations are more likely to rate their health poorly. Other than that, we found an individual's social participation can also be a significant factor. There is a need to have a more holistic approach towards the health of older adults in India. Awareness and availability of proper care and facilities are required. Policies focusing on the social, mental, and physical well-being of an individual are required to promote healthy ageing.

Our finding is in concordance with prior research literature and shows that main determinants such as mental, physical and social health are the most important factors in achieving better SRH. Our results will also be important for policymakers in allocating and planning appropriate human resources for India’s older adults and elderly as the share of this population is increasing in India over time. Our finding might also encourage other researchers to explore a new perspective which can be utilized in the best strategic development and interventions to achieve better SRH through its determinants.

## Data Availability

International Institute for Population Sciences was the nodal agency for collecting LASI Wave-1 data on behalf of the Ministry of Health and Family Welfare, Government of India. The de-identified version of the LASI Wave-1 data is publicly available to the researchers and policymakers upon formal request to the International Institute for Population Sciences for access (link to the data request document LASI_DataRequestForm_0.pdf (iipsindia.ac.in) and link for the other information for LASI data set LASI Wave-I | International Institute for Population Sciences (IIPS) (iipsindia.ac.in).

## Abbreviation

SRH: Self-rated health
LASI: Longitudinal Ageing Survey in India
AOR: Adjusted Odds Ratio
WHO: World Health Organisation
ADL: Activities of Daily Living
IADL: Instrumental Activities of Daily Living
SC: Scheduled Caste
ST: Scheduled Tribe
OBC: Other Backward Classes
NOA: None of the Above
CIDI-SF: Composite International Diagnostic Interview- Short Form
